# Viewing Ambiguous Social Interactions Increases Functional Connectivity between Frontal and Temporal Nodes of the Social Brain

**DOI:** 10.1523/JNEUROSCI.0870-20.2021

**Published:** 2021-07-14

**Authors:** Matthew Ainsworth, Jérôme Sallet, Olivier Joly, Diana Kyriazis, Nikolaus Kriegeskorte, John Duncan, Urs Schüffelgen, Matthew F. S. Rushworth, Andrew H. Bell

**Affiliations:** ^1^MRC Cognition and Brain Sciences Unit, University of Cambridge, Cambridge, United Kingdom, CB2 7EF; ^2^Department of Experimental Psychology, University of Oxford, Oxford, United Kingdom, OX2 6GG; ^3^Wellcome Centre for Integrative Neuroimaging, University of Oxford, Oxford, United Kingdom, OX3 9DU; ^4^Inserm, Stem Cell and Brain Research Institute U1208, Université Lyon 1, 69500 Bron, France; ^5^Zuckerman Mind Brain Institute, Columbia University, New York, New York, NY 10027

**Keywords:** face-processing, fMRI, monkey, social cognition

## Abstract

Social behavior is coordinated by a network of brain regions, including those involved in the perception of social stimuli and those involved in complex functions, such as inferring perceptual and mental states and controlling social interactions. The properties and function of many of these regions in isolation are relatively well understood, but less is known about how these regions interact while processing dynamic social interactions. To investigate whether the functional connectivity between brain regions is modulated by social context, we collected fMRI data from male monkeys (*Macaca mulatta*) viewing videos of social interactions labeled as “affiliative,” “aggressive,” or “ambiguous.” We show activation related to the perception of social interactions along both banks of the superior temporal sulcus, parietal cortex, medial and lateral frontal cortex, and the caudate nucleus. Within this network, we show that fronto-temporal functional connectivity is significantly modulated by social context. Crucially, we link the observation of specific behaviors to changes in functional connectivity within our network. Viewing aggressive behavior was associated with a limited increase in temporo-temporal and a weak increase in cingulate-temporal connectivity. By contrast, viewing interactions where the outcome was uncertain was associated with a pronounced increase in temporo-temporal, and cingulate-temporal functional connectivity. We hypothesize that this widespread network synchronization occurs when cingulate and temporal areas coordinate their activity when more difficult social inferences are being made.

**SIGNIFICANCE STATEMENT** Processing social information from our environment requires the activation of several brain regions, which are concentrated within the frontal and temporal lobes. However, little is known about how these areas interact to facilitate the processing of different social interactions. Here we show that functional connectivity within and between the frontal and temporal lobes is modulated by social context. Specifically, we demonstrate that viewing social interactions where the outcome was unclear is associated with increased synchrony within and between the cingulate cortex and temporal cortices. These findings suggest that the coordination between the cingulate and temporal cortices is enhanced when more difficult social inferences are being made.

## Introduction

Primates live in complex social environments with large, hierarchically organized groups. Maintaining relationships within these groups impacts on individuals' fitness (Schülke et al., 2010) and may require the ability to understand the intentions and predict the actions of other individuals within the group.

Recent research has identified brain regions that appear specialized for social cognition and reflect the complexity of a species' social environment (Kudo and Dunbar, 2001; Dunbar and Shultz, 2007). These regions range in function and complexity. Lateral prefrontal and inferior temporal regions are involved in the perception of social cues, such as facial expressions, body postures, and vocalizations (Kanwisher et al., 1997; McCarthy et al., 1997; Scalaidhe et al., 1999; Downing et al., 2001; Pinsk et al., 2005; Hadj-Bouziane et al., 2008; Tsao et al., 2008a,b; Bell et al., 2009, 2011; Diehl and Romanski, 2014). By contrast, regions concentrated within the medial frontal and orbitofrontal cortices (including the ACC), the temporoparietal junction, and subcortical regions (e.g., NAc, amygdala) are involved in complex aspects of social cognition, such as evaluating social rewards (Aharon et al., 2001; Rudebeck et al., 2006; Izuma et al., 2008; Azzi et al., 2012), monitoring the performance of conspecifics (Behrens et al., 2008, 2009; Yoshida et al., 2011, 2012), and encoding the intentions of others (Saxe and Kanwisher, 2003; Saxe et al., 2004; Wagner et al., 2012, 2016; Haroush and Williams, 2015; Wittmann et al., 2018).

At rest, these social regions have remarkably conserved patterns of functional connectivity in humans and rhesus macaques (Mantini et al., 2011; Mars et al., 2013; Sallet et al., 2013). However, there is growing interest in how these regions interact to form networks specialized for social behavior. Sliwa and Freiwald (2017) contrasted responses in the monkey brain to both social interactions between conspecifics and interactions between inanimate objects. They identified a large “social interaction network” that included regions of frontal, parietal, and temporal cortices as well as subcortical areas (caudate and amygdala).

In the human, Arioli and Canessa (2019) proposed a “social interaction perception” network based on the overlapping characteristics of networks associated with action observation (Gallese et al., 1996; Rizzolatti and Sinigaglia, 2010) and mentalizing (Koster-Hale and Saxe, 2013; Molenberghs et al., 2016). This network includes many of the regions mentioned above, most notably the posterior superior temporal sulcus (STS), temporoparietal junction, medial frontal cortex, and amygdala.

Clarifying the properties of social brain networks is of great interest, as they have been linked to clinically relevant disruptions to social behavior: for example, autism spectrum disorders (Hull et al., 2016), schizophrenia (Ebisch et al., 2018; Viviano et al., 2018), and social anxiety disorder (Liao et al., 2010; Rabany et al., 2017; Zhu et al., 2017). However, most descriptions of social networks have focused on either individual nodes or the networks at rest. A recent exception examined connectivity between the ACC and amygdala, and revealed context-dependent changes in γ and β frequency synchrony during social decisions (Dal Monte et al., 2020). This underlines the importance of social context on functional connectivity between social nodes. However, it remains unclear how communication across a wider network of regions changes during the perception of other's social interactions.

Here we address two questions concerning neural responses to social interactions in the monkey brain: (1) how activations and network interactions are affected when viewing social interactions, and crucially, (2) how these dynamics change with respect to the nature of these interactions.

We collected fMRI data from monkeys while they freely viewed videos of social interactions between nonhuman primates. These included situations where the context was clear (e.g., aggressive, affiliative) and ambiguous (e.g., 2 animals approaching each other but where the outcome was uncertain). Using fMRI in awake, behaving monkeys freely viewing videos allowed us to explore changes in functional connectivity between a moderate number of brain regions revealed to be responsive to social stimuli and to better understand how they function as a network.

## Materials and Methods

All procedures were conducted under licenses granted by the United Kingdom Home Office per the United Kingdom Animals (Scientific Procedures) Act of 1986, after approval from the University of Oxford local ethical review panel and the United Kingdom Home Office Animal Inspectorate. All husbandry and welfare conditions followed the guidelines of the European Directive 2010/63/EU for the care and use of laboratory animals.

### 

#### Animals

Three adult male monkeys (*Macaca mulatta*, M1, M2 and M3), purpose-bred within the United Kingdom, were used in the study. The monkeys were 7-8 years old and weighed 10-13 kg at the time of data collection. All monkeys lived in large communal rooms (but with separate housing areas) with several other macaques with whom they could visually interact. Monkeys M1 and M2 were pair-housed, whereas M3 was singly housed. All monkeys were kept on a 12 h light/dark cycle and were given free access to water on nontesting days, and at least 14 h access to water on testing days. Veterinary staff and animal technicians performed regular health and welfare assessments, including formalized behavioral monitoring. Before fixation training, all monkeys were implanted with MR-compatible polyether ether ketone head-posts (Rogue Research) and ceramic screws (Thomas Recordings) under aseptic conditions (for further details, see Bell et al., 2009; Chau et al., 2015).

#### Experimental setup

Stimulus presentation, reward delivery, and eye calibration were controlled using PrimatePy, an implementation of PsychoPy (Peirce, 2007) modified for primate research (Joly et al., 2014; Baumann et al., 2015). Stimuli were projected onto a screen placed 19 cm in front of the monkeys. Eye position was recorded using an MR-compatible camera (Medical Research Council Systems), and horizontal and vertical eye positions were downsampled to 25 Hz and stored for offline analysis along with reward delivery timings and TR pulse count.

#### Stimuli and data collection

**Figure 1.**
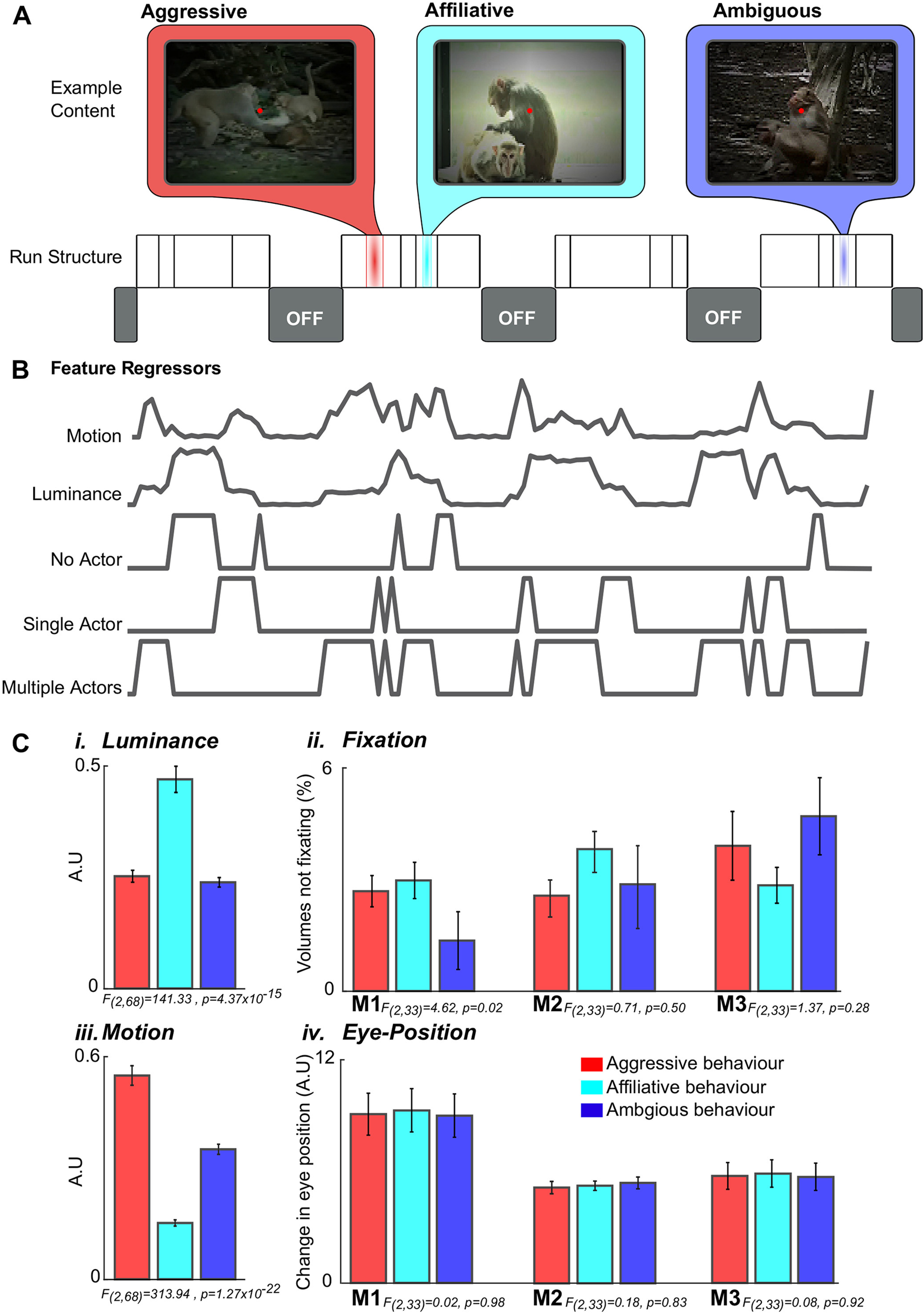
Video structure, features of interest, and low-level confounds. ***A***, Individual runs consisted of 4 video sequences interleaved with periods of blank. Each video sequence consisted of 5 to 20 s long clips of macaques engaged in social and nonsocial behaviors. Social behaviors were classified as aggressive interactions (red), affiliative interactions (cyan), or ambiguous behavior (blue). In each video sequence, periods with several immediately abutted video clips alternated with 20 s blank periods (labeled “OFF”). ***B***, Example regressors used in a GLM analysis to localize visual and social activity in the brain. Regressors were calculated from the video content and included visual features (video clips ON/OFF, luminance, and motion) and social features (number of macaques present in each scene). Note regressors shown before convolution with the hemodynamic response function. ***Ci***, Low-level confounds. ***Cii***, Average luminance. ***Ciii***, Percentage of volumes discarded from whole-brain GLM and dynamic connectivity analysis for each subject (M1-M3). ***Civ***, The average motion energy. The mean change in eye position in degrees of visual angle calculated as the Euclidean distance between adjacent samples for each subject (M1-M3) for each of the three behaviors of interest (aggressive affiliative, and ambiguous). Errorbars denote the SEM.

We used four different 220 s video sequences (total video length 880 s) presented in separate runs. There were between three and four runs per daily session for each sequence. Each video sequence consisted of 16 clips of 5, 10, or 20 s long, interspersed with 20 s blank sections. A fixation cue (red circle, 0.3 degrees) was visible during both video clips and blank periods of the sequences (see Fig. 1*A*). Throughout the sequences, monkeys were rewarded for maintaining their gaze within the boundaries of the videos (largest dimension set to 13 degrees).

Clips used in the video sequences depicted either conspecifics or the Bonnet macaque (*Macaca radiata*), a closely related species within the *Macaca* genus. The clips were obtained from three sources: two nature documentaries and a set of clips filmed within a local breeding center. Although most video clips depicted *M. mulatta*, the minority of videos depicting *M. radiata* (19% of the videos containing monkeys) were included to provide a greater range of behaviors. *M. mulatta* and *M. radiata* sometimes form mixed species social groups (Fooden, 2000). We observed no difference in the behavior of the 3 subjects when they viewed scenes containing either species, and so clips from both species were treated equally in all subsequent data analysis.

Clips featuring monkeys had either 1 or 2+ actors engaging in different social behaviors. They were classified into three different categories: aggressive, affiliative, and ambiguous (Movies 1, 2, 3). The videos obtained from the local breeding center were initially classified by staff at that facility and later confirmed by the authors. Videos obtained from the nature documentaries were classified by the authors. Clips classified as aggressive featured at least two actors clearly engaged in fighting, chasing, or otherwise aggressive behaviors. Clips classified as affiliative featured at least two actors engaged in grooming, mounting, or “hugging” behaviors. Clips classified as ambiguous also featured at least two actors pacing around one another but not necessarily in contact with one another, and not engaging in behaviors that could be easily categorized as one of the other two categories. Clips containing behaviors that did not fall into one of these three categories (e.g., eating, sleeping, etc.) were not included in further analysis. Additional control clips without macaques were included to provide a baseline for visual activation. These clips featured scenes of both natural landscapes and the interior of the breeding facility.

Movie 1 Example video stimuli showing a social interaction classified as aggressive. 10.1523/JNEUROSCI.0870-20.2021.video.1

Movie 2 Example video stimuli showing a social interaction classified as affiliative. 10.1523/JNEUROSCI.0870-20.2021.video.2

Movie 3 Example video stimuli showing a social interaction classified as ambiguous. 10.1523/JNEUROSCI.0870-20.2021.video.3

Excluding blank video sections, the breakdown of video content presented each session was as follows: clips featuring aggressive social interactions 15%, clips featuring affiliative social interactions 16%, and clips featuring ambiguous social interactions 14%. In addition, 14% of video clips featured only a single macaque, and 18% of video clips did not feature macaques. The remaining 23% of video clips featured macaques engaged in behaviors that could not be classified (see above). For each category of behavior, the percentage of content featuring *M. radiata* was as follows: 24% for clips of aggressive behaviors, 16% affiliative, 15% ambiguous, and 22% of clips featuring only a single animal. All videos were classified by human observers, and we did not include any categorization training or behavioral discrimination task for the monkeys. Thus, we cannot know for certain that monkeys would categorize the stimuli in the same way. Clips were presented in fixed orders within sequences. To ensure that to no one behavior could predict another, the clips were arranged pseudo-randomly such that all combinations of behavior (e.g., aggressive behavior preceding ambiguous behavior) occurred with equal probability.

To preserve the integrity of the video stimuli, luminance and motion energy were not altered before presentation. Instead, we quantified these low-level features (for measures of luminance and motion according to video category, see Fig. 1*C*) and included them as nuisance regressors. Two repeated-measures ANOVAs were conducted to examine differences in low features (luminance and motion) between the three behavioral classes: between-subject factor: monkey (three levels, Monkeys M1-M3), and within-subject factor: social interactions (three levels, affiliative/aggressive/ambiguous). This analysis revealed a significant main effect of both luminance (*F*_(2,68)_ = 141.33, *p* = 4.37 × 10^−15^) and motion (*F*_(2,68)_ = 313.94, *p* = 1.27 × 10^−22^); therefore, time-series for both were included as nuisance regressors in both the whole-brain GLM (see Fig. 3) and subsequent functional connectivity analyses.

Before data collection, all monkeys were trained using alternative video footage consisting of sporting events. During data collection, M1 participated in 12 sessions (total volumes: 18,040), M2 in 12 sessions (total volumes: 16,720), and M3 in 11 sessions (total volumes: 15,400).

#### MRI data acquisition

Imaging data were collected using a 3T MR scanner and a four-channel phased-array receive coil in conjunction with a radial transmission coil (Windmiller Kolster Scientific). Both fMRI images and proton-density-weighted reference images were collected while awake animals were head-fixed in a sphinx position in an MR-compatible chair (Rogue Research). fMRI data were acquired using a gradient-echo T2* EPI sequence with 1.5 × 1.5 × 1.5 mm resolution, 32 ascending slices, TR = 2 s, TE = 29 ms, flip angle = 78. Proton-density weighted images using a gradient-refocused echo sequence (TR = 10 ms, TE= 2.52 ms, flip angle= 25) were acquired as reference for body motion artifact correction during preprocessing. T1-weighted MP-RAGE images (0.5 × 0.5 × 0.5 mm resolution, TR = 2500 ms, TE = 4.01 ms, 3-5 sequences per image) were acquired from each of the 3 monkeys in separate scanning sessions and were collected under general anesthesia (for further details of anesthesia protocols and T1 image acquisition, see Mitchell et al., 2016; Ainsworth et al., 2018).

#### Data analysis

##### fMRI preprocessing

Initial fMRI data preprocessing was conducted on a run-by-run basis using Matlab toolboxes developed to correct for common artefacts in monkey functional imaging (Offline Sense and Align EPI toolboxes, Windmiller Kolster Scientific, Fresno, USA). Data were first reconstructed offline from raw image files using SENSE reconstruction to reduce Nyquist/ghost artefacts (Kolster et al., 2009). Nonlinear motion artefacts in the data were corrected on a slice-by-slice basis using a third order polynomial to align all volumes within a run to an ideal EPI reference image (Kolster et al., 2014).

Further preprocessing of the reconstructed and motion-corrected data was conducted using functions from both AFNI (Cox, 1996) and FSL (fMRI of the Brain Software Library) (Jenkinson et al., 2012). Individual runs were concatenated to yield a single 4D data file for each session and the resultant data were skull-stripped and signal outliers were removed (using 3dDespike from the AFNI package) (Cox, 1996). Remaining volumes that were contaminated by excessive motion were identified based on the volume-to-volume variance (Power et al., 2012) (performed with fsl_motion_outliers using the dvars option). For each session, individual volumes with variance greater than the session mean + 2.5 times the session SD were identified as outliers and modeled in further analysis as nuisance regressors. For each monkey, the average percentage of volumes per session identified in this way were M1 4 ± 2%, M2 6 ± 1%, and M3 7 ± 1%.

Data were registered to the NMT standard monkey atlas (Seidlitz et al., 2018) with a two-step registration process. First, the mean EPI image for each session was registered to the relevant monkey's high-resolution T1-weighted structural image. This was achieved by boundary-based registration of mean images, with field maps used to simultaneously correct for EPI field distortions (Jenkinson and Smith, 2001; Jenkinson et al., 2002; Greve and Fischl, 2009). Each monkey's T1 structural image was then registered to the NMT template image with 9 degrees of freedom. For each session, the two relevant transformation matrices were combined and saved for further analysis. Segmentation of T1 structural images to generate gray, white matter, and CSF masks was achieved using FAST (Zhang et al., 2001), and masks for each monkey were transformed into EPI space for use in further analysis. Finally, during initialization of the GLM (for details, see below), fMRI data were spatially smoothed (3 mm FWHM), temporally filtered (3 dB cutoff 100s), and intensity normalized.

##### Video feature regressors

Regressors coding for visual and social features of the stimuli were created on a frame-by-frame basis from the content of the videos (examples shown in Fig. 1*B*). First, a binary video ON/OFF regressor was created in which ones corresponded to frames with video content and zeros for frames during blank fixation periods. Two additional regressors were created based on the overall luminance and total motion of each frame of the video. Total motion between video frames was calculated using a block matching method with video frames divided into 25 × 25 pixel blocks (Block Matching function, Computer Vision System Toolbox, MATLAB 2014a, The MathWorks). These two additional regressors were treated as nuisance regressors to remove the effect of these low-level features on the data. Finally, three binary regressors were created based on the number of monkeys visible on each frame (no monkeys, 1 monkey, and 2 or more monkeys). Video content was manually scored on a frame-by-frame basis and assigned to the appropriate regressor. All regressors were downsampled to 0.5 Hz to match the 2 s TR of the fMRI sequence. Before convolution with a γ function (SD 1.5 s, mean lag 3 s), all regressors were modified such that volumes in which the monkey failed to fixate for >80% of that volume were set to zero.

##### Whole-brain GLM analysis

**Figure 2.**
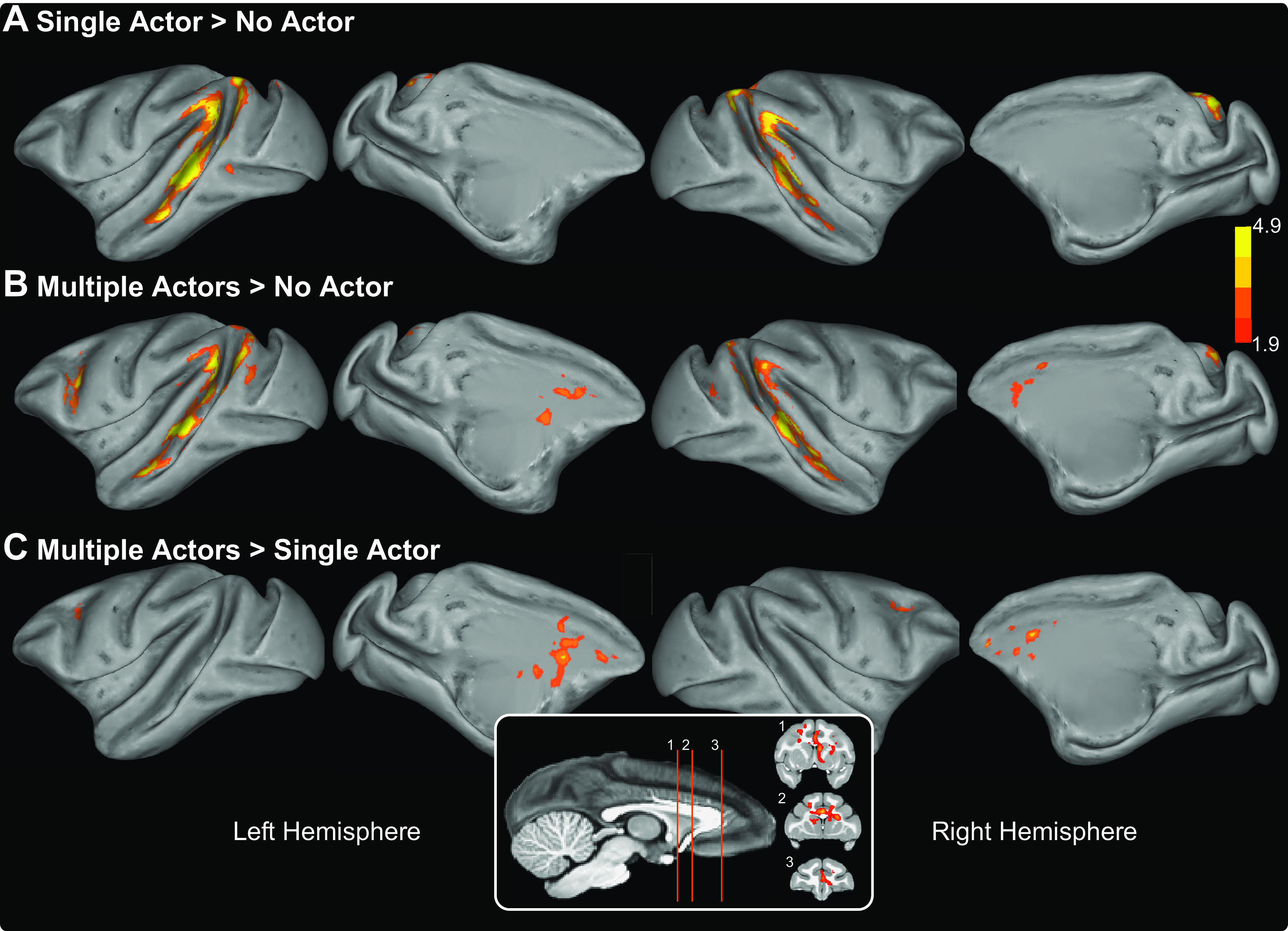
Cortical activation on viewing single actors and multiple actors engaged in natural behavior. ***A-C***, Inflated brains showing significant clusters from three contrasts derived from the number of actors visible in the videos. All data presented are from the third-level GLM analysis that combined activations from all 3 animals. The contrasts include scenes with a single actor versus scenes with no actors visible (***A***), scenes containing multiple actors versus scenes with no actors visible (***B***), and scenes containing multiple actors versus scenes containing single actors, regardless of the behavior of the visible actors (***C***). Medial frontal lobe activation from the multiple versus single actor contrast is shown as insets overlaid on coronal anatomic slices. All data shown survived a cluster correction at *z* statistic > 1.9 and *p* < 0.05.

**Figure 3.**
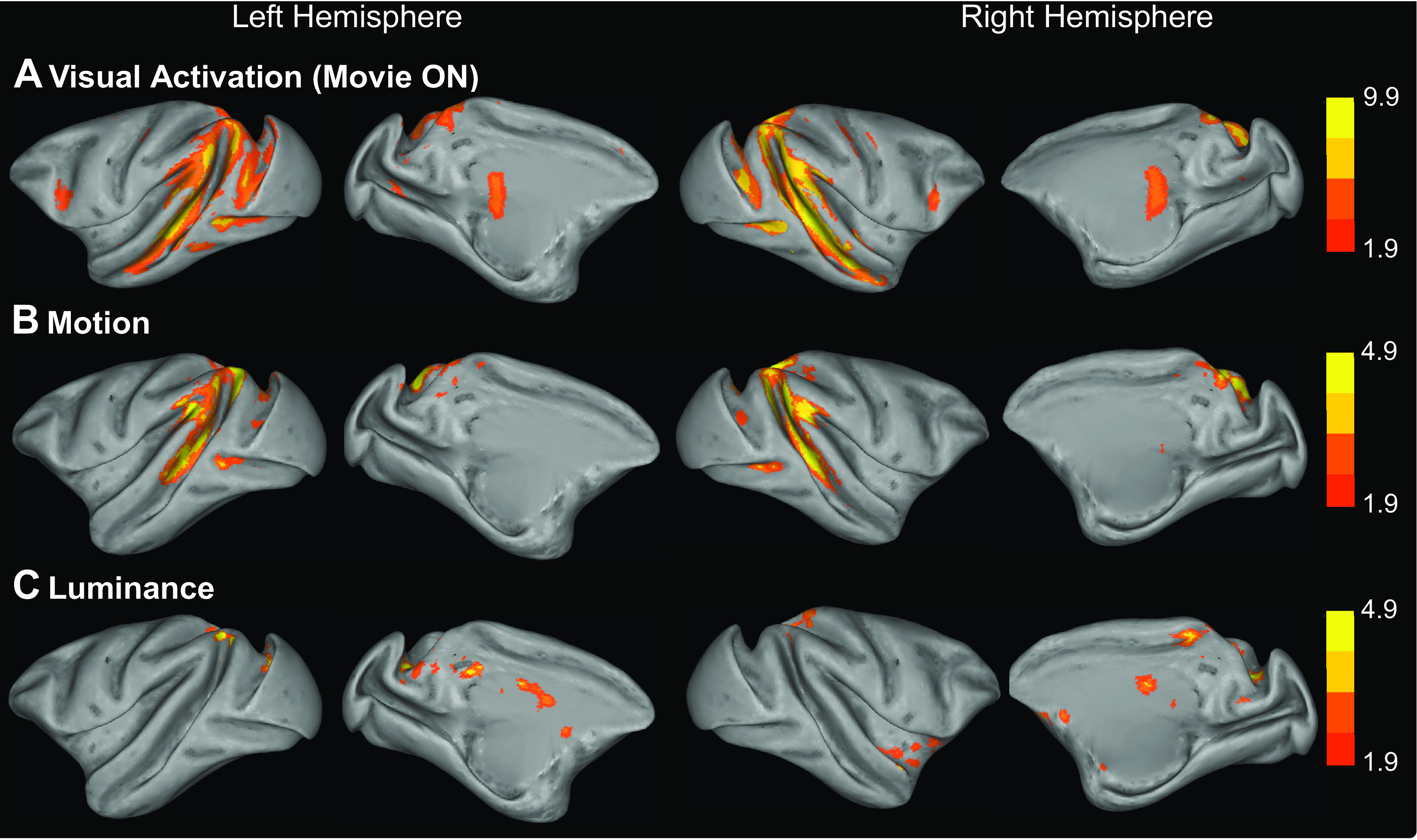
Cortical activation associated with low-level visual video features. ***A-C***. Inflated brains showing significant clusters from three contrasts of low-level visual features calculated from the videos. All data presented are from the third-level GLM analysis combining activation from all 3 animals. The contrasts include the following: the basic visual activation during each session (video ON/OFF, ***A***); the motion within the video, calculated by a block matching algorithm examining differences between frames of the video content (see Materials and Methods for details, ***B***); and the luminance of the video scenes (***C***). Note the differences in scales as different thresholds (*z* statistic > 6.5 *z* statistic > 1.9 and *z* statistic > 1.9) were applied to the data shown in ***A-C***, respectively, and all images were cluster-corrected at *p* < 0.05.

We conducted an initial analysis to identify brain regions that respond selectively depending on the number of actors (see Fig. 2). We used a multilevel, univariate GLM analysis using the FSL FEAT tool (Woolrich et al., 2004). The first-level GLM in this analysis was conducted on the processed 4D fMRI data of each session. As noted, the model at this level included nuisance regressors for low-level visual features (video ON/OFF, total motion between video frames and overall luminance; see Fig. 3) as well as the regressors of interest (number of actors, 0, 1, or multiple). Three contrasts of interest were included in the model to identify areas of the brain activated by viewing differing numbers of animals: one actor versus no animals, multiple actors versus no animals, and multiple versus single actors. In addition, individual regressors were included in the model for each volume identified as being contaminated by excessive motion using fsl_motion_outliers (described above). The results from the first-level analyses were then combined in three, second-level mixed-effects GLMs (FLAME 1 and 2), corresponding to one for each monkey. We then combined these into a third final group-level fixed-effects GLM (Woolrich et al., 2004). Significant clusters were identified from the *z* statistic images using a threshold of z > 1.9 and cluster correction of *p* < 0.05.

##### Social network ROI definition

**Figure 4.**
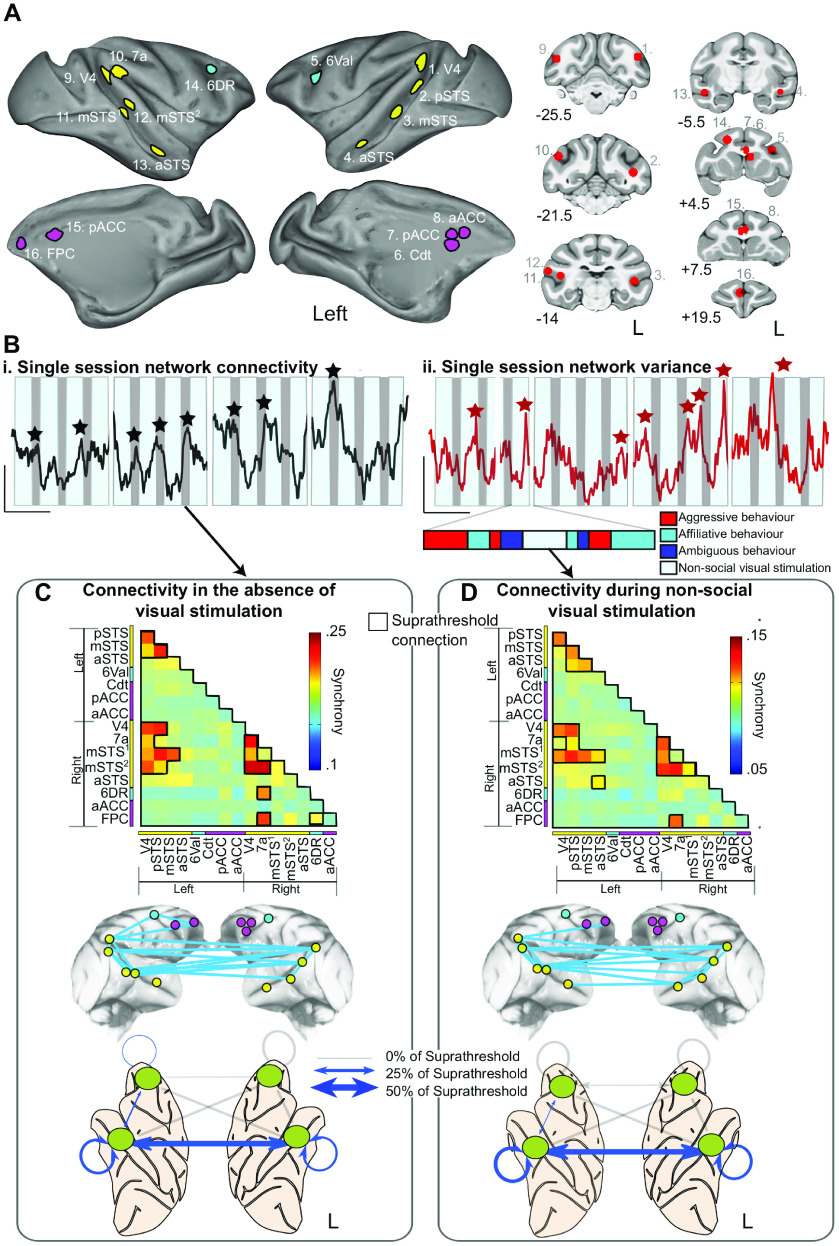
The structure and dynamic functional connectivity of the putative social network. ***A***, Surface maps and coronal slices showing the 16 ROIs selected from the *z* statistic maps in Figure 2 as constituting the core of a social network. Magenta represents medial frontal cortex. Cyan represents lateral PFC. Yellow represents temporal/parietal ROIs. Exact ROI coordinates are available in Extended Data Figure 4-1. ***B***, Single-session examples of the global dynamics of the network. The average dynamic functional connectivity between ROIs in the network, calculated using a time-windowed phase synchrony measure (top, black trace; calibration: 100 s, 0.5 AU) and the average variance in functional connectivity within the network (bottom, red trace; calibration: 100 s, 0.1 AU). Both examples were averaged over the 880 s of unique video content presented in a single session. The ON/OFF structure of the video is shown behind each trace (movie ON/OFF represented by light blue/gray bars, respectively). Interruptions in the bars represent the stop/start of each of the four individual runs. Stars represent peaks in mean network connectivity and mean variance in connectivity, respectively. ***C***, ***D***, Detailed analysis of the structure of the putative social network in the absence of visual stimulation (***C***) and during nonsocial visual stimulation (***D***). Functional connectivity matrices (top) show the strength of all possible connections between ROIs during both these conditions. Suprathreshold connections (the strongest 15% of connections, outlined in black) were selected from both matrices and the anatomic properties of the connections visualized with two network schematics. In the first schematic, suprathreshold connections (shown in light blue) are displayed, linking the relevant ROIs (colored according to the above scheme) of the core network (middle). In addition, suprathreshold connections are summarized in a simplified representation linking the left and right frontal and temporal lobes. The thickness of the connection between these lobes corresponds to the proportion of the total suprathreshold connections, which are present between the lobes (bottom).

ROIs within our putative social network were defined based on the activation clusters for two contrasts: single > no monkeys and multiple > no monkeys. Local maximal voxels were identified within the clusters obtained from each of these contrasts. The 16 most active voxels from the temporal (9 voxels) and frontal (7 voxels) lobes were selected. These were converted to 16 spherical ROIs with a diameter of 4 mm, distributed across the two hemispheres (8 per hemisphere; for ROI locations, see Fig. 4*A*; Extended Data Fig. 4-1). We chose to focus on only 16 ROIs as this gave a reasonable distribution of well-sized ROIs across frontal and temporal cortex, without including spurious clusters. ROIs were masked according to the LV-FOA-PHT cytoarchitectonic standard atlas (Van Essen et al., 2012) such that each ROI was constrained to a single cortical area and there was no overlap between adjacent ROIs.

##### Calculation of dynamic functional connectivity

Before the calculation of dynamic functional connectivity between ROIs, the mean BOLD signal from all sessions from each ROI was filtered using a GLM incorporating two confound time-series: one generated from the CSF mask and another derived from timestamps denoting the onset of the reward pulses. The residual BOLD time-series obtained from this model was used for the subsequent analysis.

Before calculating phase synchrony, we performed six additional one-way ANOVAs examining differences in fixation and change in gaze position between the three behavioral classifications (each with one factor, three levels; affiliative/aggressive/ambiguous; see Fig. 1*C*). This analysis revealed a significant main effect of fixation for M1 (*F*_(2,33)_ = 4.62, *p* = 0.021) but not for M2 (*F*_(2,33)_ = 0.71, *p* = 0.50) or M3 (*F*_(2,33)_ = 1.37, *p* = 0.28). By contrast, there was no main effect of gaze direction for M1 (*F*_(2,33)_ = 0.02, *p* = 0.98), M2 (*F*_(2,33)_ = 0.18, *p* = 0.83), or M3 (*F*_(2,33)_ = 0.08, *p* = 0.92). To ensure that changes in dynamic functional connectivity were not driven by differences in behavior of the subjects, change in gaze location and fixation was included as a nuisance regressor when calculating phase synchrony between ROIs (see below). These regressors were created by downsampling offline behavioral data to 0.5 Hz (again matching the TR of the MRI sequence before convolving the resultant time-series with a γ function (SD 1.5 s, mean lag 3 s).

Dynamic functional connectivity was assessed by the pairwise calculation of relative phase synchrony between all ROIs (Rosenblum et al., 1996). In contrast to correlation-based measures of functional connectivity, relative phase synchrony provides a measure of coherence unbiased by the amplitude of the signals. However, phase synchrony measures are sensitive to the frequency content of paired signals. Previous studies have considered both within (1:1) and across frequency (1:n) phase synchrony (Palva et al., 2005). In this study, no assumptions were made about specific frequency functional connectivity, and phase synchrony was calculated from 0.01 to 0.5 Hz. Fourier analysis of the BOLD time-series revealed peaks evident at 0.02 and 0.04 Hz, but all frequencies in the aforementioned range are considered in all subsequent analyses.

As with previous dynamic functional connectivity studies, phase synchrony was calculated for short overlapping windows of paired time-series. The length of sliding windows is typically limited by decreased signal-to-noise ratio and increased variability as window length decreases (Hutchison et al., 2013), whereas others have suggested that a minimum window size of 33 s is required to reveal stable modular architecture within the brain (Jones et al., 2012). Comparable window lengths have been used in previous dynamic functional connectivity studies of resting state activity (C. Chang et al., 2013; Hutchison et al., 2013). We therefore calculated relative phase synchrony between the instantaneous phase of each pair of signals over a 32 s time window. To ensure that the subsequent phase synchrony was calculated with sufficient temporal resolution to reveal changes linked to events within the videos, each window was offset by 2 s and overlapping the adjacent window by 30 s. Phase synchrony time-series were subsequently filtered using a GLM to remove low-level features (luminance, motion, and change in gaze location) and to account for volumes with poor behavioral performance (subject fixation). All synchrony values for each session were arcsine-transformed to account for any values at the extremes. Finally, the normalized and cleaned synchrony values were averaged across repeated viewings of the videos to yield a time course corresponding to the complete 14.8 min of unique video content.

##### Statistical analysis of dynamic functional connectivity

Before analyzing dynamic functional connectivity within our network in response to different social behaviors, we first validated the technique. Initially, we examined global functional connectivity within the network over the time course of the scanning sessions by calculating the mean functional connectivity and mean variance across all pairwise connections in the network. We then calculated the mean strength of each connection during periods of noninterest (blank periods in the video and nonsocial content) and used a threshold selecting for the strongest 15% of connections to view the structure of the network.

**Figure 5.**
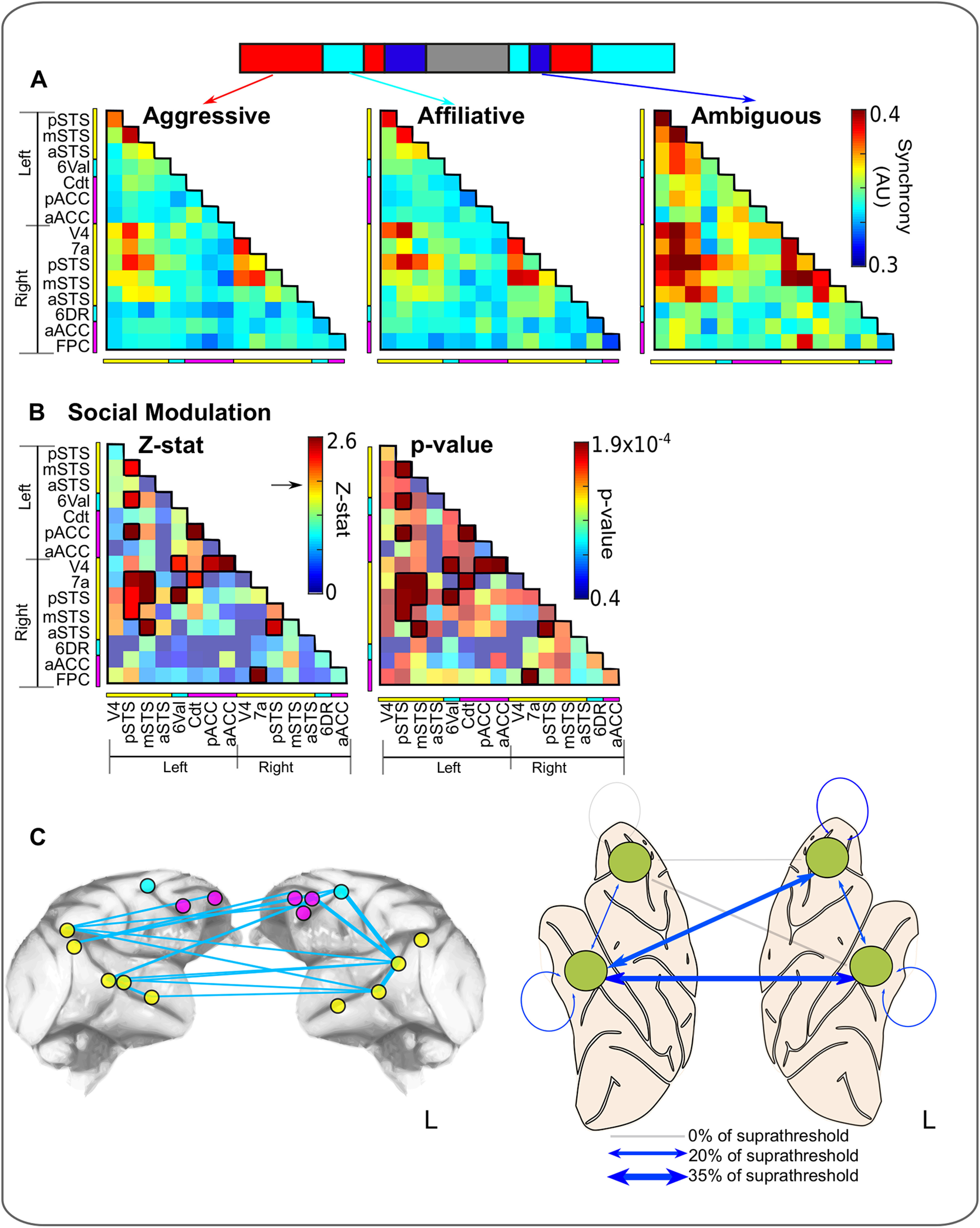
Social modulation of network functional connectivity. ***A***, Average connectivity matrices calculated from scenes during which monkeys viewed multiple macaques engaged in each of the three social interactions of interest (aggressive, affiliative, and ambiguous behavior). ***B***, Results of a repeated-measures ANOVA assessing the degree to which network functional connectivity was modulated by social interaction (within-subject factor with three levels, aggressive/affiliative/ambiguous; for details, see Materials and Methods). The *z* statistic and *p* values obtained from this analysis for each connection are displayed as summary matrices. Connections with the strongest social modulation were selected with a threshold of *z* > 2.05 (equivalent to the strongest 15% of connections, suprathreshold connections outlined in black). ***C***, Suprathreshold connections are graphically represented in blue between ROIs in the network (left). Simplified graphical representation of connections between the left and right frontal and temporal lobes. The thickness of the connecting line represents the proportion of suprathreshold connections displaying social modulation of functional connectivity.

To assess the extent to which viewing different social interactions modulated network functional connectivity, we averaged the phase synchrony values for each pairwise connection between ROIs within the network on a session-by-session basis for three different network states. These states corresponded to the manually scored time courses for scenes containing multiple actors engaged in three different behaviors: (1) affiliative behavior (e.g., lip smacking, grooming behavior, etc.); (2) aggressive/dominant behavior (e.g., piloerection, teeth baring, and/or physical confrontation); and (3) ambiguous behavior in which the nature of interactions between the two or more actors was unclear (average functional connectivity matrices for each state shown in Fig. 5*B*).

A repeated-measures ANOVA was conducted for each connection using these average functional connectivity values with one between-subject factor: monkey (three levels, Monkeys M1-M3), and one within-subject factor: social interactions (three levels, affiliative/aggressive/ambiguous). The degrees of freedom and *p* value calculated for each connection from this analysis are displayed in Figure 5*B*. For visualization and to ensure consistency with the previous whole-brain analyses, *p* values were converted to *z* statistics, resulting in a single matrix with a *z* statistic for each connection. From this matrix, only connections with a *z* statistic > 2.05 (representing the strongest 15% of total connections) were considered for further analysis.

To determine essential nodes in the network, two measures were calculated from the resulting binary matrix of socially modulated connections using the Brain Connectivity Toolbox (Rubinov and Sporns, 2010). First, for each ROI, the degree or number of connections to the ROI was calculated. Second, the importance of each ROI was assessed by calculating eigenvector centrality. Eigenvector centrality is biased toward well-connected nodes. Therefore, ROIs with high eigenvector centrality are not only well connected within a network but have a lot of connections to other well-connected ROIs.

To explicitly link changes in functional connectivity to the specific types of behavior, an additional analysis was conducted in which changes in functional connectivity were examined after the onset of clips containing each of the three behavior types (aggressive, affiliative, or ambiguous behavior). We aligned 11 s segments of phase synchrony time-series (4 s preclip to 6 s postclip onset) with a 2 s delay to allow for the hemodynamic lag. The aligned time-series were then interpolated using a cubic spline and averaged from the strongest 15% of connections between five anatomic groupings: cingulate-cingulate connections, cingulate-temporal connections and temporo-temporal connections, premotor-cingulate connection, and premotor-temporal connections (including connections within and across hemispheres). Two tests were conducted on the resulting time-series. First, to identify statistically significant increases in synchrony after video onset, we performed a series of one-tailed, one-sample *t* tests. These *t* tests compared the phase synchrony for each behavior type against baseline. Second, to identify which behavior was associated with the strongest phase synchrony, a GLM was used to directly contrast the phase synchrony values associated with each of the three behaviors. This GLM therefore included three contrasts: ambiguous > (affiliative + aggressive); affiliative > (ambiguous + aggressive); and aggressive > (ambiguous + affiliative). Significant *p* values obtained from both the *t* tests and GLM were corrected for multiple comparisons using Bonferroni corrections.

## Results

To characterize the relationships between regions of the monkey brain involved in processing social interactions, we presented videos containing conspecific and visually similar and closely related but non-rhesus macaque (*M. radiata*) actors to 3 rhesus macaques while collecting BOLD fMRI data. The videos consisted of 5-20 s clips interspersed with blank periods (Fig. 1*A*). Each clip contained monkey actors engaged in natural behavior with the number, identity, and behavior of the actors as well as the scene location changing randomly between clips (for additional detail, see Materials and Methods). All 3 monkeys were rewarded for maintaining their gaze within the borders of the video but were allowed free eye movement within this limit. On average, the 3 monkeys maintained this level of fixation for 90 ± 3% (M1), 89 ± 7% (M2), and 62 ± 8% (M3) of presented video content for each session.

### Regions in the primate brain responsive to social behaviors

A univariate GLM analysis was conducted to identify regions in the brain that selectively respond to social stimuli (see Materials and Methods). This model included regressors based on low-level visual features (ON/OFF, luminance, and motion) as well as regressors scoring the number of actors on the screen (Fig. 1*B*).

When monkeys viewed scenes with only single actors visible, we observed strong bilateral activation in the temporal cortex (Fig. 2*A*; single actor > no actor, *z* statistic > 1.9, cluster-corrected, *p* < 0.05). This activation followed the fundus of the STS. Within this sulcus, three semi-distinct clusters were arranged along the anterior-posterior direction and extended onto both the superior and inferior banks of the sulcus. No activation was evident outside the temporal cortex.

By contrast, when monkeys viewed scenes featuring more than one actor regardless of their behavior, strong activation was observed in both the frontal and temporal cortices (multiple actors > no actors; Fig. 2*B*). Within the temporal cortex, activation was again bilateral and closely matched the STS clusters observed when monkeys viewed scenes containing single actors. Activation within the frontal lobe was less extensive and limited to two discrete clusters. The larger cluster extended bilaterally along the cingulate gyrus, whereas the smaller cluster was located around the spur of the arcuate sulcus in the left premotor cortex.

Directly contrasting the responses for single versus multiple actors (regardless of behavior) revealed strong activation within the cingulate cortex (multiple actors > single actor; Fig. 2*C*). Specifically, this contrast showed bilateral activation within the cingulate gyrus that extended in the left hemisphere into the caudate nucleus. In addition, directly contrasting multiple > single actors revealed bilateral activation on the posterior bank of the arcuate sulcus (*z* statistic > 1.9, cluster-corrected *p* < 0.05).

The three regressors of no-interest that accounted for the low-level visual features (video onset/offset, motion, and luminance) predictably elicited strong activation along both banks of the STS and, to a lesser extent, in the tertiary visual areas (Fig. 3, note differing scales).

### Dynamic functional connectivity within a social network

The above data show that activation of frontal areas was more prominent when monkeys viewed scenes containing multiple monkeys. We therefore examined the functional interactions, in the form of dynamic functional connectivity, between frontal, temporal, and subcortical regions corresponding to instances where monkeys viewed the different social behaviors. We did so using a progressive four-stage analysis approach, and we present the results of each stage to clearly illustrate how the final result was achieved.

Briefly, we assessed the suitability of this approach for identifying how social behaviors affect global functional connectivity within the network by averaging connectivity measures across all ROIs and social behaviors (Stage 1). Next, we examined changes to individual pairwise functional connections in response to nonsocial stimuli (Stage 2) followed by changes in response to any of the social behaviors (Stage 3). Finally, we examined what specific behaviors elicited the most notable changes to pairwise functional connections within the network (Stage 4).

#### Global changes in response to social behaviors

We first defined a “putative social network” consisting of 16 ROIs from the clusters identified in the previous analysis. These ROIs were centered on the maximally responsive voxels identified within the contrasts of interest above and included locations distributed across the frontal, temporal, and parietal lobes. In the left hemisphere, four ROIs were identified in the temporal lobe, including area V4, and three ROIs located along the STS. In addition, four ROIs were identified within the left frontal lobe and subcortex, including one in premotor cortex (area 6Val), two in the cingulate gyrus (area 24a/b), and one in the caudate nucleus.

Although ROIs in the right hemisphere followed a broadly similar distribution, there were some notable differences in the location of ROIs in the left and right hemispheres. In the right hemisphere, four ROIs were identified in the temporal lobe; this included an ROI in V4 as well as three ROIs located along the STS. In addition, a single ROI was located in the parietal cortex of the right hemisphere (the inferior aspect of area 7a). Finally, three ROIs were located in the right frontal cortex. These included one premotor ROI (area 6DR), one in the cingulate gyrus (24a/b), and one ROI in frontopolar area 10 (Fig. 4*A*; see Extended Data Fig. 4-1).

10.1523/JNEUROSCI.3192-20.2021.f4-1 Figure 4-1 Putative social network ROIs. Table containing the CARET atlas cortical region, and centroid coordinate in NMT space for each putative social network ROI. Download Figure 4-1, XLSX file.

We calculated the dynamic functional connectivity (see Materials and Methods) between all possible pairings of ROIs in this network over an entire session. After preprocessing and concatenating the individual BOLD time-series for each run, the time-series were averaged over the sets of videos that were repeated within a session. We then examined the changes in functional connectivity relative to both the visual and social features of the 880 s video sequence (for more details, see Materials and Methods).

We first focused on two measures of functional connectivity within this network: (1) changes in the average connectivity strength, calculated across all pairwise functional connections within the putative social network; and (2) changes in the variance in connectivity, again using the same approach.

Both measures varied considerably over the time course of the videos with sharp, transient increases in both average functional connectivity and variance (Fig. 4*Bi*). Peaks in the average network connectivity were generally time-locked with periods during which the monkeys were required to maintain fixation in the absence of visual stimulation (black markers). By contrast, peaks in network variance occurred predominantly during periods of visual activation (Fig. 4*Bi*,*Bii*, red markers).

This latter observation raises the possibility that either the visual and/or social content of the video clips was associated with changes in functional connectivity across a smaller number of specific connections, rather than a more uniform network-wide change in functional connectivity (which would have presumably increased the average connectivity, but not the variance).

#### Specific changes to pairwise connections in response to nonsocial stimuli

We therefore performed a similar analysis, this time focusing on specific functional connections within the network. This analysis revealed that certain periods were marked by selective increases in specific functional connections within the network (Fig. 4*C*,*D*). During blank periods (no video clips), the network was dominated by temporo-temporal functional connections (Fig. 4*C*). Suprathreshold connections (which we have here defined as the strongest 15% of all pairwise functional connections) were primarily interhemispheric, temporo-temporal functional connections (44% of suprathreshold connections), followed by within-hemisphere connections in both right and left temporal cortices (22% and 17% of suprathreshold connections, respectively, as illustrated in Fig. 4*C*).

By contrast, functional connectivity within the frontal cortex was less pronounced (accounting for 17% of all suprathreshold connections). These connections were exclusively intrahemispheric, and included connections both within the right frontal lobe as well as those linking the right frontal and temporal cortices (5% and 11%, respectively; Fig. 4*C*), while no connections involving the left frontal lobe were found to be suprathreshold.

During periods with nonsocial video clips (visual scenes lacking any monkey actors), network functional connectivity was again dominated by temporo-temporal functional connections (Fig. 4*D*). These primarily included interhemispheric temporo-temporal functional connections (44% of suprathreshold connections) followed again by within-hemisphere functional connections in both right and left temporal cortices (28% and 22% of suprathreshold connections, respectively). Again, connections involving frontal regions were less affected (only 6% of suprathreshold connections involved areas of the frontal lobe) with the only suprathreshold connections being those linking frontal and temporal cortex in the right hemisphere, as illustrated in Figure 4*D*. No intrahemispheric fronto-frontal functional connections were suprathreshold.

#### Frontal-temporal functional connectivity is modulated by social content

To assess changes in network functional connectivity associated with viewing specific behaviors, we used a repeated-measures ANOVA consisting of one between-subject factor: monkey (three levels, Monkeys M1-M3), and one within-subject factor of interest: social interactions (three levels aggressive/affiliative/ambiguous; for details, see Fig. 5; Materials and Methods). For this analysis, we applied a statistical threshold (*z* > 2.05) to the matrix of *z* statistics for social interactions so as to focus on connections of interest.

This analysis revealed that social behavior was associated with the modulation of both fronto-temporal and temporo-temporal functional connections (Fig. 5). This included intrahemispheric fronto-temporal functional connections, which accounted for 18% of total suprathreshold connections (6% and 12% for left and right intrahemispheric fronto-temporal connections, respectively). Interhemispheric fronto-temporal functional connections (linking the left frontal to right temporal lobe) accounted for an additional 29% of total suprathreshold connections. Temporo-temporal functional connections accounted for a further 47% of total suprathreshold connections. Suprathreshold tempo-temporal connections were predominantly interhemispheric connections (35% of total suprathreshold connections), with only a limited number of left and right interhemispheric temporo-temporal connections (both 6% of total suprathreshold connections). By contrast, fewer fronto-frontal suprathreshold connections were found to be modulated by social information. Fronto-frontal connections within the left hemisphere accounted for 6% of total suprathreshold connections. There were no suprathreshold fronto-frontal connections within the right hemisphere or the between the left and right hemispheres.

**Figure 6.**
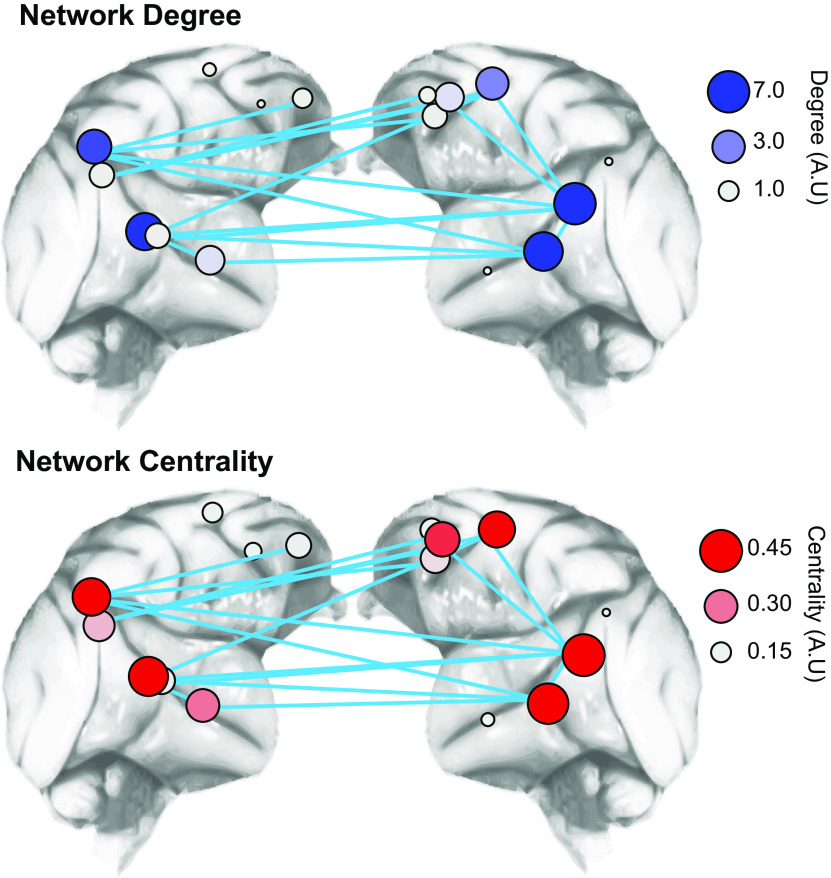
Network degree and eigenvector centrality of suprathreshold socially modulated connections. Graphical representation of degree (top) and eigenvector centrality (bottom) of each ROI calculated from suprathreshold socially modulated connections. ROIs with greater degree (blue nodes) and centrality (red nodes) indicated as larger and stronger colored nodes within the network. The positions of ROIs are approximate, and all ROIs are shown on lateral cortical surface.

To further examine which specific ROIs were linked by suprathreshold, socially modulated connections, we calculated two metrics: degree centrality and eigenvector centrality, from connectivity matrices summarizing the social modulation of each connection (Fig. 6). These were used to quantify how “central” each ROI is to the network (Fig. 6). Both measures use an ROI's connectivity to indicate its importance to a network; an ROI's degree centrality simply reflects the sum of its connections, whereas an ROI's eigenvector centrality gives greater weights to nodes connected to other well-connected nodes (for more detail, see Materials and Methods).

This analysis revealed a clear distinction within the temporal lobe. Middle temporal ROIs exhibited both a greater degree (therefore more likely to be linked with suprathreshold social modulated connections) and greater centrality (more likely to be connected to other ROIs with a high number of socially modulated connections) than anterior temporal ROIs (aSTS) or ROIs located in V4. Although there was a clear hemispheric difference in the frontal lobe, there was no other clear distinction in ROI network centrality or degree within the frontal lobe. While ROIs within the cingulate gyrus (notably those in the left hemisphere) exhibited both high network degree and centrality scores, the same was true for the premotor cortex ROIs (particularly the left premotor cortex ROI).

#### Ambiguous scenes elicit increased functional connectivity in fronto-temporal connections

The above data demonstrate that functional connectivity, both within the temporal lobe and between the frontal and temporal lobes, is modulated by the nature of social behavior viewed by a monkey. This analysis does not, however, reveal which specific behavioral types contained in the video sequences (aggressive, affiliative, or ambiguous behavior) were associated with the observed changes in connectivity, a vital question.

**Figure 7.**
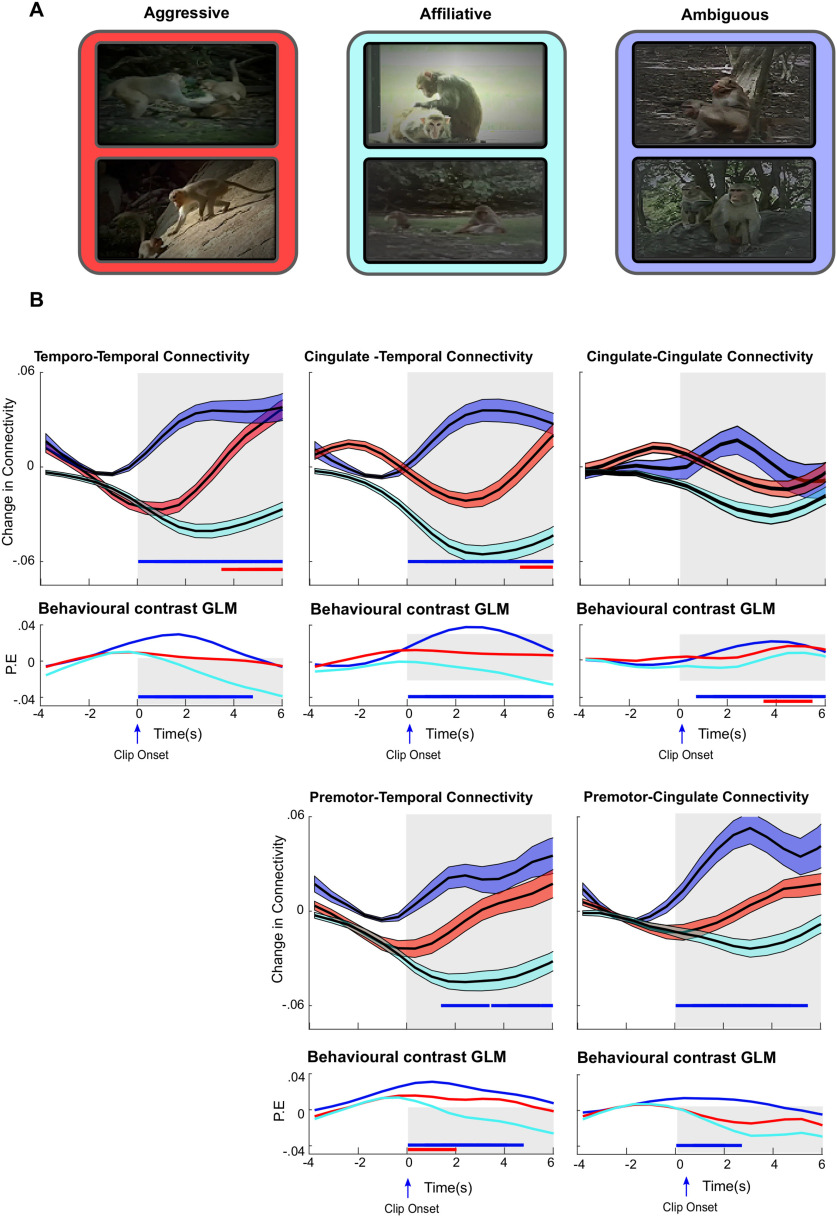
Viewing ambiguous behavioral interactions drives increased functional connectivity between cingulate gyrus and temporal lobe. The average time course of functional connectivity between the cingulate gyrus and premotor cortex and temporal lobe ROIs aligned to the onset of clips in which the behavioral interactions were classified as aggressive (red), affiliative (cyan), or ambiguous (blue). ***A***, Example frames of the three behaviors contained in the clips. ***B***, Top subplots, The clip-onset triggered functional connectivity calculated from temporo-temporal (top left panels), cingulate-temporal (top middle panels), cingulate-cingulate (top right panels), premotor-temporal (bottom middle panels), and premotor-cingulate (bottom right panels) connections for each of the three behaviors viewed. Consistent with previous analyses, only the strongest 15% of connections were considered. Colored bounds denote the SEM. Top subplots, Colored bars represent time points with functional connectivity significantly stronger than pre-onset baseline (significance was determined by one-sample, one-tailed, *t* test *p* < 0.05). Bottom subplots for each panel show parameter estimates for three contrasts: ambiguous > the average of affiliative and aggressive behavior (blue); aggressive > the average of affiliative and ambiguous behavior (red); and affiliative > the average of aggressive and ambiguous behavior (cyan). Bottom subplots, Colored bars represent time points at which the relevant contrast is significant at *p* < 0.05. All *p* values corrected for multiple comparisons using Bonferroni correction.

Previous work has shown how it is possible to link specific changes in network correlations with specific scenes in a video (Hasson et al., 2004; Russ and Leopold, 2015). We used a similar approach to examine how functional connectivity changes in response to the three types of behavior (Fig. 7*A*).

We aligned the average phase synchrony between ROIs in the frontal and temporal lobes to the onset of the video clips and normalized these values to the prior baseline to show relative changes in functional connectivity (see Materials and Methods). As the previous analysis indicated the existence of socially modulated functional connections to both cingulate and premotor ROIs, we explicitly averaged functional connections between both these sets of ROIs and the temporal lobe (Fig. 7*B*).

This analysis, shown in Figure 7, revealed differences in functional connectivity within our network depending on the specific behavior viewed. First, temporo-temporal and cingulate-temporal functional connectivity was significantly higher than baseline over the first several seconds of video clips where the behavior was classified as ambiguous (Fig. 7*B*). In addition, viewing video clips in which the behavior was classified as aggressive was associated with an increase in temporo-temporal and cingulate-temporal functional connectivity, albeit 4-6 s after video clip onset. However, directly contrasting the functional connectivity associated with viewing each of the three behaviors using a GLM (see Methods and Materials for details) revealed that both temporo-temporal and cingulate temporal functional connectivity was significantly stronger during the first 5 s after video onset when animals viewed video clips featuring ambiguous as opposed to affiliative or aggressive behavior (Fig. 7*B*, ambiguous > aggressive + affiliative, blue parameter estimate).

Consistent with the previous results, premotor-temporal and premotor-cingulate functional connectivity was significantly higher than baseline over the first 5 s of video clips where the behavior was classified as ambiguous (Fig. 7*B*, bottom panels). Directly contrasting functional connectivity for each of the three behaviors confirmed the importance of viewing ambiguous behavior. Both premotor-temporal and premotor-cingulate functional connectivity after video onset was significantly higher when animals viewed video clips featuring ambiguous as opposed to affiliative or aggressive behavior (Fig. 7*B*, ambiguous > aggressive + affiliative, blue parameter estimate).

By contrast, cingulate-cingulate functional connectivity did not increase significantly over baseline when animals viewed any of the three behaviors (Fig. 7*B*). However, further analysis did reveal that cingulate-cingulate functional connectivity was significantly higher when animals viewed ambiguous behavior as opposed to aggressive or affiliative behavior 1-6 s after video onset.

Finally, viewing video clips in which the behavior was classified as affiliative did not cause a significant increase in functional connectivity between temporal, cingulate, or premotor ROIs. Furthermore, functional connectivity viewing affiliative video clips was not significantly higher than functional connectivity when viewing aggressive or ambiguous behaviors (affiliative > aggressive + ambiguous, Fig. 7*B*, cyan parameter estimate).

## Discussion

We have examined how regions of the male monkey brain responsive to social stimuli coordinate their activities in response to dynamic and complex social interactions. Monkeys were shown short video clips involving one or more monkey actors and which broadly fell into one of three categories: affiliative, aggressive, and a third category where the nature of the interaction was uncertain (e.g., clips of one actor approaching another, or two actors approaching one another). Viewing clips of social interactions activated several brain regions that were concentrated within the frontal and temporal lobes (Fig. 2). Critically, although temporal lobe regions were activated regardless of the number of actors, frontal areas were most prominently recruited while monkeys viewed clips with more than one actor. Functional connectivity across this social brain network, and in particular between the frontal and temporal regions, varied according to the behavior viewed by the monkeys. Furthermore, significant increases in functional connectivity between cingulate gyrus and temporal and premotor ROIs were observed when monkeys viewed interactions labeled as ambiguous (Fig. 7). We propose that the increase in cingulate-temporal lobe functional connectivity associated with viewing ambiguous social interactions may reflect the increased neural processing necessary to make accurate predictions about upcoming behaviors that are unnecessary or reduced when subjects view more predictable interactions.

Here, we first present how our selection of nodes in our social network relates to preexisting literature before discussing why certain social contexts may lead to increased functional connectivity between nodes in this network.

### Composition of a social network

Recent advances in the field of social cognition, including in the nonhuman primate brain, have led to a better level of understanding of the individual roles for these regions (Platt et al., 2016). For our purposes, we selected a subset of brain regions based on which areas were reliably activated by our stimuli using our imaging protocols. We grouped these regions into a “putative social network.” The majority, if not all, the regions in our putative social network are already well established as being involved in social cognition.

To begin, our social network included a number of ROIs distributed along the STS. These ROIs presumably correspond to the canonical face-selective regions (Tsao et al., 2008a; Bell et al., 2009; Pinsk et al., 2009). Although we did not independently assess the boundaries for body-part selective regions, they are typically located immediately adjacent to face-selective regions (Bell et al., 2009; Pinsk et al., 2009), and so we can assume our STS regions incorporate body-part selectivity as well.

The contribution of these regions to our social perception task may seem self-evident. After all, these regions have been shown to be reliably activated by a number of social features, including the presence and discrimination of faces and facial expressions (Haxby et al., 2000; Hadj-Bouziane et al., 2008; Bell et al., 2009; Furl et al., 2012; Morin et al., 2015), biological motion (Perrett et al., 1985; Jastorff et al., 2012), as well as more complex aspects of social perception and cognition (Fisher and Freiwald, 2015; McMahon et al., 2015; Park et al., 2017; Shepherd and Freiwald, 2018). Further, recent studies have shown the STS to be sensitive to the composition and hierarchy of the monkey actors, not just their identity (Sallet et al., 2011; Noonan et al., 2014), which may be particularly relevant to the evaluation of social interactions. Therefore, it is possible that, in addition to participating in the “simple” visual processing and reconstruction of the images (e.g., extracting the visually derived semantic codes as proposed by Bruce and Young, 1986), these regions are playing a more complex role in evaluating nonvisual identity-derived semantic features of the actors, such as their degree of dominance and role in the social interaction.

Our network also included a single parietal ROI, located in the right area 7a. A number of recent studies have identified area 7a as a component of networks involved in processing social information. For example, Schurz et al. (2020) have suggested that human inferior parietal lobule is a component of an empathy network. Furthermore, Sliwa and Freiwald (2017) identified an ROI within area 7a as a constituent of their “exclusive social interaction” network, although the location of our area 7a ROI is both more anterior and inferior of that identified by Sliwa and Freiwald (2017).

In addition, our network included bilateral premotor cortex ROIs, both of which were located on the posterior bank of the arcuate sulcus, albeit with subtle hemispheric differences in location. In the left hemisphere, the premotor ROI was located on the inferior aspect of the arcuate sulcus within area 6Val, while the right premotor cortex ROI was more superior, centered on area 6. These ROIs are notable as premotor cortex is increasingly being implicated in social cognition. For example, detailed single-neuron recordings have revealed subpopulations of mirror neurons in premotor area F5 that respond to actions essential for judging social hierarchy and status, including gaze direction (Coude et al., 2016) and lip smacking (Ferrari et al., 2003). Furthermore, Sliwa and Freiwald (2017) recently demonstrated significant overlap between two contrasts: one mapping social interactions and the other localization of the mirror neuron system, arguing that this overlap indicated a role for mirror neurons in processing social intentions of an interaction as well as simple motor understanding of the interaction.

Our putative social network included a number of ROIs located in or adjacent to medial frontal cortex, including within the cingulate and frontopolar cortices. The three ROIs located in the cingulate cortex have a role in social cognition in both humans and nonhuman primates. In particular, the cingulate gyrus has been shown to be central to social valuation (Rudebeck et al., 2006; S. W. Chang et al., 2013; Apps and Ramnani, 2014). Adjacent areas of cingulate cortex and other medial frontal regions have also been implicated in tracking the behavior and intentions of other agents (Yoshida et al., 2011, 2012; Haroush and Williams, 2015; Hill et al., 2016; Wittmann et al., 2016; Fatfouta et al., 2018; Lockwood and Wittmann, 2018). The frontopolar cortex ROI, located within area 10m, appears compatible with the activation “exclusive to social interactions” observed by Sliwa and Freiwald (2017).

Finally, we observed strong activation in the caudate nucleus. While we cannot speculate what specific role caudate may serve in interpreting social behavior, the area's contribution to social cognition in general is becoming clearer. It has recently been associated with the default mode network (Alves et al., 2019), which itself has been linked to the social brain (Mars et al., 2012). In humans, responses to monetary and social rewards have been associated with striatal activity (Izuma et al., 2008). In monkeys, feedback for self versus others is discriminated by striatal neurons (Baez-Mendoza and Schultz, 2013; Baez-Mendoza et al., 2016). Closer to our protocol, Sliwa and Freiwald (2017) found that caudate was activated during viewing of social scenes; and Noonan et al. (2014) found that the volume of gray matter in the caudate covaried with social status in macaques.

There were a few notable absences in the list of regions reliably activated by our social stimuli, namely, the amygdala and face-responsive regions in the anterior temporal lobe and PFC. To be clear, we do not propose that these areas were not involved in the processing of our social stimuli. Instead, we believe their lack of activation in our particular study may be the result of technical issues and/or experimental design. For example, in the case of the amygdala, which has a well-documented role in social cognition, there are at least two possible explanations for the lack of significant activation in our study. The first is related to the fact that the amygdala can be difficult to image in monkeys, particularly larger male monkeys who have extensive musculature on either side of their skulls. This additional muscle mass increases the distance between the receiver coil elements and the target structure, thus inhibiting signal detection. The degree to which this is an issue is, of course, related to the precise placement and size of the coil elements. However, we note that previous studies that have used either the same experimental setup (Chau et al., 2015) or similar study design (Sliwa and Freiwald, 2017) have found amygdala activity; and so it may have been a function of our animals and coil positioning. We compared the temporal signal-to-noise ratio from the amygdala to ROIs within the frontal (area 24) and temporal lobe (area TEa) (data not shown) and found that while the mean TSNR was not significantly different across the ROIs, the variance of the TSNR across sessions was much larger for the amygdala ROIs. This suggests that, across session, the quality and stability of the signal obtained from the amygdala were reduced compared with ROIs closer to the cortical surface.

A second potential explanation is that, while the amygdala may have been active during our task, it was equally active across all conditions; and thus, no significant differences were observed in our contrasts. This would fit with more modern studies of amygdala function that highlight its role in generalized linking of social and nonsocial stimuli to outcomes rather than being, for example, a “fear module” (i.e., selectively activated by specific social interactions, contexts, or stimuli). Analysis of the average percent signal change for basic contrasts of interest (e.g., multiple vs single macaques; data not shown) for amygdala ROIs did indeed reveal it to be much weaker relative to frontal and temporal ROIs. However, given the previously described variation between sessions, this finding is not conclusive; and thus, both explanations may contribute to the lack of significant effects in the amygdala in this study.

In the case of the PFC, technical limitations are less likely (as signal is more reliably obtained from these superficial cortical regions); and so, it is possible that the lack of significant activation is somehow because of our experimental design. Again, we cannot say for certain, but we offer the following hypothesis: there are two key features of our design that are different from most other studies that have found significant activation in PFC in response to faces and other social stimuli. First, in other studies, monkeys are typically explicitly rewarded for fixating faces, making face stimuli particularly important for behavior. Second, they were usually the only stimuli present for the animals to look at (Scalaidhe et al., 1999; Tsao et al., 2008b). This was not the case with our study. Faces were just part of the complex scenes being presented, and the monkeys were not explicitly required to track the faces for reward. Further, it has been argued by the coauthors that these regions within PFC (including lateral orbital sulcus) are less concerned with face-processing per se, and more concerned with linking choices (as represented by any type of stimuli, including but not limited to faces) and outcomes (Chau et al., 2015; Lopez-Persem et al., 2020; Sallet et al., 2020). It is thus conceivable that while there might have been activation to face stimuli (over no actors) in frontal cortex, it did not achieve a sufficient signal-to-noise ratio to surpass our statistical threshold.

### Role of frontal recruitment in interpreting social interactions

The most notable observation in our study was the increased functional connectivity of the cingulate cortex to frontal and temporal regions while monkeys observed interactions that were not clearly affiliative or aggressive. In the latter two video types, the monkeys viewing the clips would not be required to make any judgments or predictions about the nature of the interaction, as it was clearly depicted in the video. On the other hand, even in a passive-viewing case, it is possible that the monkeys viewing ambiguous interactions might automatically attempt to infer the ultimate outcome of the actors' behavior.

In this scenario, one interpretation is that inferring the consequences or outcomes of ambiguous social behavior, even without an active task component, not only requires regions of the brain where neurons encode the social features of stimuli (e.g., face patches in the inferior temporal cortex) but also areas in the cingulate cortex. This hypothesis is consistent with recent electrophysiological studies of cingulate cortex in nonhuman primates. These studies have revealed how neurons within the cingulate cortex encode a range of information essential for making social decisions, including shared reward experience (S. W. Chang et al., 2013) and predictions of other animals' future decisions (Haroush and Williams, 2015), while regions in the medial frontal cortex have been shown to encode the actions of other animals (Yoshida et al., 2011). Furthermore, recent research has revealed that synchrony between neurons of the cingulate cortex and other brain areas (in this instance, the amygdala) can be modulated by social context (Dal Monte et al., 2020). Dal Monte et al. (2020) revealed that coherence between neuronal activity recorded from the cingulate cortex and amygdala was increased when animals shared a reward but decreased when only self-rewarded. However, it should be noted that these studies detail cingulate activity while nonhuman primates were required to make decisions based on social cues or information, rather than observing social interaction between other animals.

Yet more evidence for the recruitment of frontal regions during the viewing of social interactions has been provided by recent fMRI studies in humans. For example, Sapey-Triomphe et al. (2017) presented point-light images of multiple actors either interacting or not interacting to participants ranging in age from 8 to 41 years of age. The participants were required to state whether the images were interacting or not. Across all age groups, STS, middle temporal gyrus, anterior temporal lobe, and inferior frontal gyrus were activated during the presentation of these social interactions. However, stronger activation was observed among adult participants in frontal, parietal, and striatal (caudate) areas. It was also these participants who were more successful at recognizing whether the point-light images were interacting or not, the implication being that the additional activation was correlated with improved social perception. Similarly, Gardner et al. (2015) demonstrated that increased familiarity with videos of subjects performing dance moves resulted in decreased correlations within an action-observation network.

Collectively, these results highlight two potential features of dynamic interactions in social networks: (1) that interactions between frontal, parietal, and temporal cortices are not static but rather change dynamically depending on the features and nature of the social stimuli being presented; and (2) making inferences or active interpretations of social interactions (rather than passive viewing of social scenes) may recruit additional frontal activation.

Our data parallel these conclusions by showing the greatest amount of coherence among nodes in our putative social network when monkeys were viewing social scenes where the outcome was unclear, compared with predictable scenes. Therefore, we speculate that this additional frontal recruitment reflects the additional cognitive demands of deciphering ambiguous social scenes.

### Future directions

This study represents an early step toward understanding the role of frontal, striatal, and temporal cortex in social cognition. Critically, our task was a passive task; the monkeys were not required to make judgments about the behavior of the actors. This limits our ability to guess what information might be passing between the frontal and temporal regions. Yet, there is an increasing number of studies examining social behavior between more than one subject (Yoshida et al., 2012; Haroush and Williams, 2015; Grabenhorst et al., 2019), so it is becoming more feasible to conduct experiments involving 2 or more monkeys interacting with one another. To fully understand how brain regions involved in social cognition interact and what type of information passes between them will likely require such experiments. Future experiments will seek to add a behavioral/decision-making component to these types of experiments, such as having the animals guess what the outcome of different ambiguous situations might be, possibly having them make predictions about upcoming interactions. This way, we may take one step closer to an understanding of the degree to which nonhuman primates exhibit rudimentary “theory of mind”-like abilities, and what role regions, such as those discussed in this study, might play in that cognitive function.

Our subject animals were all male; and given the significant ecological differences related to gender, it would be premature to assume our results extrapolate directly to female macaques. It would be fascinating to contrast our results with similar results obtained from female macaques, macaques of different ages, and macaques at different levels within a single social hierarchy.

In conclusion, fMRI is limited in its ability to reveal the nature of information being passed from one region to another. This technique can identify circuits of interest to study with a more suitable method that can clarify the nature of the millisecond-by-millisecond information being passed between nodes in a complex cortical network. For example, how are the new information requirements in situations when animals are viewing ambiguous social interactions communicated to/from frontal and temporal regions? How are the neural representations within temporal cortex of the actors being updated or modulated while the scene plays out? Moreover, fMRI cannot reveal much about the causal role of regions within brain networks. These are questions that are better addressed using techniques with, for example, better temporal resolution than MRI, and the ability to interfere with brain function (e.g., lesions and inactivations). No doubt, such experiments will yield exciting new insights as to the role of interactions between frontal, temporal, and striatal regions in social cognition.
